# Long-Term Effects of Moderate Concussive Brain Injury During Adolescence on Synaptic and Tonic GABA Currents in Dentate Granule Cells and Semilunar Granule Cells

**DOI:** 10.3389/fnins.2022.800733

**Published:** 2022-03-14

**Authors:** Akshay Gupta, Laura Dovek, Archana Proddutur, Fatima S. Elgammal, Vijayalakshmi Santhakumar

**Affiliations:** ^1^Department of Pharmacology, Physiology and Neuroscience, Rutgers New Jersey Medical School, Newark, NJ, United States; ^2^Department of Molecular, Cell and Systems Biology, University of California, Riverside, Riverside, CA, United States

**Keywords:** dentate gyrus, traumatic brain injury, tonic GABA current, development, adolescence, synaptic inhibition

## Abstract

Progressive physiological changes in the hippocampal dentate gyrus circuits following traumatic brain injury (TBI) contribute to temporal evolution of neurological sequelae. Although early posttraumatic changes in dentate synaptic and extrasynaptic GABA currents have been reported, and whether they evolve over time and remain distinct between the two projection neuron classes, granule cells and semilunar granule cells, have not been evaluated. We examined long-term changes in tonic GABA currents and spontaneous inhibitory postsynaptic currents (sIPSCs) and in dentate projection neurons 3 months after moderate concussive fluid percussion injury (FPI) in adolescent rats. Granule cell tonic GABA current amplitude remained elevated up to 1 month after FPI, but decreased to levels comparable with age-matched controls by 3 months postinjury. Granule cell sIPSC frequency, which we previously reported to be increased 1 week after FPI, remained higher than in age-matched controls at 1 month and was significantly reduced 3 months after FPI. In semilunar granule cells, tonic GABA current amplitude and sIPSC frequency were not different from controls 3 months after FPI, which contrast with decreases observed 1 week after injury. The switch in granule cell inhibitory inputs from early increase to subsequent decrease could contribute to the delayed emergence of cognitive deficits and seizure susceptibility after brain injury.

## Introduction

Traumatic brain injury (TBI) leads to diverse consequences including impaired memory and reasoning, depression, anxiety as well as enhanced risk for epilepsies and Alzheimer’s disease (LoBue et al., 2019; Faden et al., 2021). Adverse outcomes after TBI can evolve over months to years, highlighting the need to understand progressive changes in cellular and circuit function (Marshall et al., 2015). The hippocampal dentate gyrus is a focus of cellular pathology and functional changes following brain injury in humans and various experimental models (Kharatishvili et al., 2006; Hunt et al., 2010; Villasana et al., 2015; Meier et al., 2016; Neuberger et al., 2017a,b; Wolf et al., 2017; Korgaonkar et al., 2020b; Parga Becerra et al., 2021). The dentate gyrus is a crucial gateway to the hippocampal circuit, serving as a locus for memory processing and as a check against excessive excitability and reentrant epileptiform activity (Dengler and Coulter, 2016). Strong synaptic and extrasynaptic inhibition of dentate granule cells (GCs) contributes to their sparse activity and is known to be disrupted in TBI and epilepsies (Peng et al., 2004; Rajasekaran et al., 2010; Pavlov et al., 2011; Gupta et al., 2012; Boychuk et al., 2016; Kahn et al., 2019; Parga Becerra et al., 2021). Studies examining injury-induced changes in inhibition in GCs, the major projection neuronal subtype, days to weeks after trauma have identified changes in synaptic and extrasynaptic GABA_*A*_ currents, which differ between injury models and with injury severity (Santhakumar et al., 2001; Pavlov et al., 2011; Gupta et al., 2012; Boychuk et al., 2016). While long-term (> 4 weeks) posttraumatic changes in GC inhibition have been examined, the results are varied, with persistent decreases in both tonic and synaptic GABA_*A*_ currents after controlled cortical impact injury (CCI) (Boychuk et al., 2016; Parga Becerra et al., 2021) and reduced synaptic inhibition, while tonic GABA_*A*_ currents remained unchanged after severe concussive trauma (Pavlov et al., 2011). We previously reported an increase in GC synaptic and tonic GABA currents 1 week after moderate concussive brain injury in adolescent rats (Gupta et al., 2012). This clinically relevant adolescent concussive injury paradigm impairs working memory performance 1–4 weeks postinjury followed by heightened risk for seizures 1–3 months after injury (Neuberger et al., 2017b; Korgaonkar et al., 2020a,b). Since GABAergic signaling, which critically regulates dentate memory function and epileptogenesis (Dengler and Coulter, 2016), undergoes developmental plasticity spanning in this period (Kapur and Macdonald, 1999; Gupta et al., 2020), we sought to determine if GC inhibition undergoes progressive changes after concussive TBI.

In addition to GCs, we reported that brain injury impacts inhibition of semilunar granule cells (SGCs), a sparse, morphologically distinct dentate projection neuron (Williams et al., 2007; Gupta et al., 2012; Afrasiabi et al., 2021). SGCs have been proposed to support the feedback inhibition of GCs needed to maintain sparse activity and contribute to cellular memory representations (Larimer and Strowbridge, 2010; Erwin et al., 2020). Interestingly, both tonic and synaptic GABA currents in SGCs are decreased 1 week after FPI, which contrasts with increases observed in GCs at the same time (Gupta et al., 2012). Since changes in tonic GABA currents enhance SGC excitability early after brain injury (Gupta et al., 2012), they could contribute to posttraumatic memory dysfunction. While tonic GABA currents in SGCs are greater than in GCs during adolescence, SGC tonic GABA currents undergo a developmental decline into adulthood (Gupta et al., 2020). Thus, whether the early decrease in SGC tonic GABA currents after injury persists at later time points when SGC tonic GABA currents have declined remains to be established. Since GCs and SGCs are proposed to play distinct roles in dentate feedback inhibition and memory processing (Larimer and Strowbridge, 2010; Erwin et al., 2020), it is crucial to understand how injury-induced changes in inhibition evolve in these dentate projection neuron subtypes. This study examined the long-term changes in tonic and synaptic GABA currents in GCs and SGCs after moderate concussive brain injury in adolescent rats to determine if early cell-specific posttraumatic changes in inhibition, observed at 1 week, are maintained at later time points.

## Materials and Methods

### Animals

All experiments were conducted under IACUC protocols approved by Rutgers-NJMS and the University of California at Riverside and conformed with the ARRIVE guidelines. Wistar rats (Charles River) aged 60–70 or 120–180 days, which were over 1 and 3 months after sham or brain injury, respectively, were used in the study. Due to the potential effects of hormonal variation on GABA currents (Maguire and Mody, 2009), recordings were restricted to male rats.

### Surgery and Fluid Percussion Injury

Lateral fluid percussion injury (FPI) was performed on adolescent (postnatal days 24–26) male Wistar rats as described previously (Dixon et al., 1987; Li et al., 2015). Briefly, under ketamine–xylazine anesthesia (25 mg/kg of ketamine and 5 mg/kg of xylazine, i.p.), a 2-mm hole was trephined on the left side of the skull (3 mm posterior to the bregma and 3.5 mm from the lateral to sagittal suture) to expose the dura, and a syringe hub with a 2.6-mm inner diameter was bonded to the skull with cyanoacrylate adhesive. One day later, animals were anesthetized with isoflurane and attached to an FPI device (Virginia Commonwealth University, Richmond, VA, United States). A pendulum was dropped to deliver a brief (20 ms) 2.0–2.2-atm impact to the intact dura resulting in moderate FPI with reproducible hilar cell loss (Gupta et al., 2012; Li et al., 2015). Sham injured animals received identical surgery and treatment, but the pendulum was not dropped.

### Slice Physiology

**FIGURE 1:**
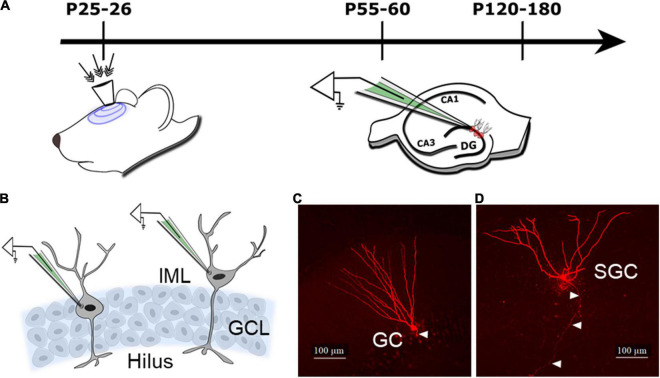
Schematic of the experimental design and cell types. **(A)** Schematic illustrates timeline of injury (indicated by arrows to the surgically implanted hub) and slice physiology in the current study. **(B)** Schematic of the dentate granule cell layer (GCL) illustrates the location of cells targeted for patch clamp recording of granule cells (GCs) in the GCL and semilunar granule cells (SGCs) in the inner molecular layer (IML). **(C,D)** Representative maximum intensity projections from confocal image stacks of a biocytin-filled GC **(C)** and SGC **(D)** from rats 1 month after sham injury. Arrowheads indicate axon.

One and 3–5 months after FPI or sham injury rats were decapitated under isoflurane anesthesia for slice physiology (Figure 1A). Horizontal brain slices (300 μm) were prepared in ice-cold sucrose-artificial cerebrospinal fluid (sucrose-aCSF) containing (in mM): 85 NaCl, 75 sucrose, 24 NaHCO_3_, 25 glucose, 4 MgCl_2_, 2.5 KCl, 1.25 NaH_2_PO_4_, and 0.5 CaCl. Slices were bisected and incubated at 32°C for 30 min in a holding chamber containing an equal volume of sucrose-aCSF and recording aCSF and were subsequently held at room temperature. The recording aCSF contained (in mM): 126 NaCl, 2.5 KCl, 2 CaCl_2_, 2 MgCl_2_, 1.25 NaH_2_PO_4_, 26 NaHCO_3_, and 10 D-glucose saturated with 95% O_2_ and 5% CO_2_ (pH 7.4). Whole-cell voltage-clamp recordings were performed at 33°C under IR-DIC visualization using MultiClamp 700B (Molecular Devices) as detailed previously (Gupta et al., 2012; Yu et al., 2016). As illustrated in the schematic (Figure 1B), small oval or teardrop-shaped cells in the granule cell layer were targeted for recording GCs, while crescent-shaped neurons located in the inner molecular layer were targeted for SGC recordings. Data were low-pass filtered at 3 kHz, digitized using DigiData 1440A, and sampling frequency using pClamp10 was acquired at 10 kHz. GABA currents were recorded using microelectrodes (5–7 MΩ) containing (in mM): 125 CsCl, 5 NaCl, 10 HEPES, 2 MgCl_2_, 0.1 EGTA, 2 Na-ATP, 0.5 Na-GTP, and 0.2% biocytin, titrated to a pH 7.25 in the absence of without added GABA or GABA transporter antagonists (Gupta et al., 2012; Yu et al., 2013). Kynurenic acid (3 mM KynA), a glutamate receptor antagonist, was used to isolate GABA currents in cells held at −70 mV. Recordings were discontinued if series resistance increased by > 20%. Baseline recordings (in KynA) were obtained for over 5 min before perfusing GABA_*A*_ receptor (GABA_*A*_R) antagonist, bicuculline methiodide (BMI, 100 μM) or gabazine (SR95531, 10 μM). Cesium-based internal solution was used to block K^+^ conductances underlying postsynaptic GABA_*B*_ currents. Custom macros in IgorPro7.0 were used to measure tonic GABA currents as the difference in baseline currents in blockers and for detecting sIPSCs (Yu et al., 2013). All salts were purchased from Sigma-Aldrich.

### Cell Morphology

All recorded slices were fixed in 4% paraformaldehyde and processed for biocytin staining using Alexa Fluor 594-conjugated streptavidin (Gupta et al., 2012; Swietek et al., 2016) for cell identification. Sections were imaged using a Zeiss LSM 510 confocal microscope for classification. Images of biocytin-filled cells (Figures 1C,D) were used to distinguish GCs with compact dendritic arbors and hilar axonal projections from SGCs with wide dendritic angle, multiple primary dendrites, and axons projecting to the hilus (Gupta et al., 2020; Afrasiabi et al., 2021). A subset of cells were reconstructed using Neurolucida 360 for illustration (Gupta et al., 2020).

### Statistics

Cumulative probability plots of sIPSC parameters were constructed by pooling an equal number of events from each cell. Wilcoxon rank test (WRT) was conducted on data that failed tests for normalcy or equal variance. Cohen’s d test was used to estimate effect size. Student’s *t*-test (SigmaPlot 12.3) was used to test for statistical differences in tonic GABA currents and one-way ANOVA was used to estimate effect of time. Sample sizes (*n* = cells/number of rats) were not predetermined and conformed with those employed in the field. Data that fell over 3 standard deviations outside the mean were considered outliers and rejected. Significance was set at *p* < 0.05. Data are reported as mean ± SEM (standard error of the mean).

## Results

### Posttraumatic Increase in Granule Cell Tonic GABAergic Currents Decline With Time

**FIGURE 2:**
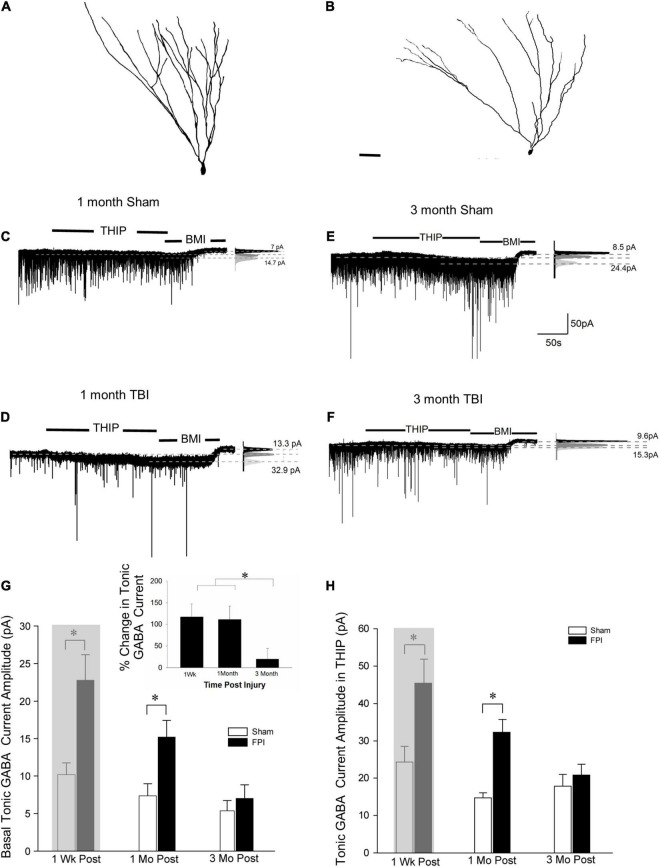
Granule cell tonic GABA current amplitude progressively declines 1–3 months after brain injury. **(A,B)** Representative Neurolucida reconstructions of GCs from rats 3 months after sham **(A)** and fluid percussion injury (FPI) **(B)**. **(C,D)** Representative traces from granule cell 1 month after sham **(C)** or FPI **(D)** illustrate tonic GABA current as the baseline current blocked by bicuculline. **(E,F)** Representative tonic GABA current traces in GCs 3 months after sham **(E)** and FPI **(F)**. Panels to the right show Gaussian fits to the positive half of histograms derived from 30-s recording periods in control conditions, in the presence of THIP (1 μM) and during the perfusion of bicuculline methiodide (BMI) used to determine tonic current. The dashed lines indicate the Gaussian means, and the difference current is noted. **(G)** Summary plots of tonic GABA current amplitude (pA) in granule cells from rats 1 week (data from Gupta et al., 2012 shown in gray box), 1 month, and 3 months after FPI. Inset: Summary of percentage change in tonic GABA current normalized to sham. **(H)** Summary plots of tonic GABA current amplitude in granule cells measured in the presence of THIP (1 μM) from rats 1 week (data from Gupta et al., 2012 shown in gray box), 1, and 3 months after FPI. *Indicates *p* < 0.05.

Tonic GABA currents mediated by extrasynaptic receptors influence GC excitability (Stell et al., 2003). While we have reported an increase in GC tonic GABA currents 1 week after moderate FPI in 25–26-day-old rats (Gupta et al., 2012), a period that parallels human adolescence in terms of neurological developmental (Semple et al., 2013; Sengupta, 2013), no changes in GC tonic GABA currents were observed 1–5 months after severe FPI in adult rats (Pavlov et al., 2011). To determine the time course of changes in tonic GABA currents after developmental FPI, we examined GCs from rats injured during adolescence at two time points: 1 and 3–4 months post-FPI. GCs were identified based on somata in the cell layer and compact dendritic arbors with one or two primary dendrites in *post hoc* biocytin fills (Figures 1C, 2A,B). Tonic GABA current amplitude in GCs remained larger than in age-matched sham controls 1 month post-FPI (Figures 2C,D, in pA, sham: 7.2 ± 1.3, *n* = 8/4; FPI: 15.2 ± 2.2, *n* = 6/5; *p* = 0.004 by *t*-test) similar to what was reported at 1 week (Figure 2G, 8.4 ± 1.1, *n* = 9; FPI: 20.8 ± 3.4, *n* = 6; *p* < 0.05 by *t*-test reported in Gupta et al., 2012, shown shaded). However, tonic GABAergic current in GCs from rats 3 to 5 months after FPI was not different from age-matched controls (Figures 2E–G, sham: 5.4 ± 1.4, *n* = 10/7; FPI: 7.0 ± 1.8, *n* = 9/6, *p* > 0.05 by *t*-test). Notably, while differences in GC tonic GABA currents were not statistically significant 1 week to 3 + months after sham injury [*F*_(2,24)_ = 1.46, *p* = 0.2], there was a decline in tonic GABA currents with time after FPI during the same period [Figure 2G, *F*_(2,18)_ = 8.9, *p* = 0.002]. These data demonstrate progressive recovery of the early increase in GC tonic GABA currents to sham control levels by 3 months.

We previously reported that THIP, a GABA_*A*_R agonist selective for δ subunit containing receptors, increased GC tonic GABA current amplitude 1 week after FPI (Gupta et al., 2012). Consistent with the progressive change in tonic GABA current amplitude after FPI, GC tonic GABA current amplitude in THIP (1μM) was also higher than in controls 1 month after FPI (in pA sham: 14.7 ± 3.0, *n* = 5/3; FPI: 32.3 ± 3.4, *n* = 5/4, *p* < 0.05 by *t*-test), but was not different from sham controls by 3 months post-FPI (Figure 2H, in pA sham: 18.9 ± 3.4, *n* = 7/5; FPI: 20.8 ± 2.8, *n* = 3/3, *p* = 0.75 by *t*-test). Once again, the effect of time after sham injury on tonic GABA currents in GCs remained stable over 1 week to 3 + months [*F*_(2,15)_ = 0.97, *p* = 0.4]. However, there was a decline in tonic GABA currents with time after FPI during the same period [Figure 2G, *F*_(2,11)_ = 4.7, *p* = 0.03]. Together, these data identify that changes in GABA_*A*_Rs with δ subunits contribute to the progressive alterations in GC tonic GABA currents after brain injury.

### Long-Term Reduction in Inhibitory Synaptic Drive to Granule Cells After Brain Injury

**FIGURE 3:**
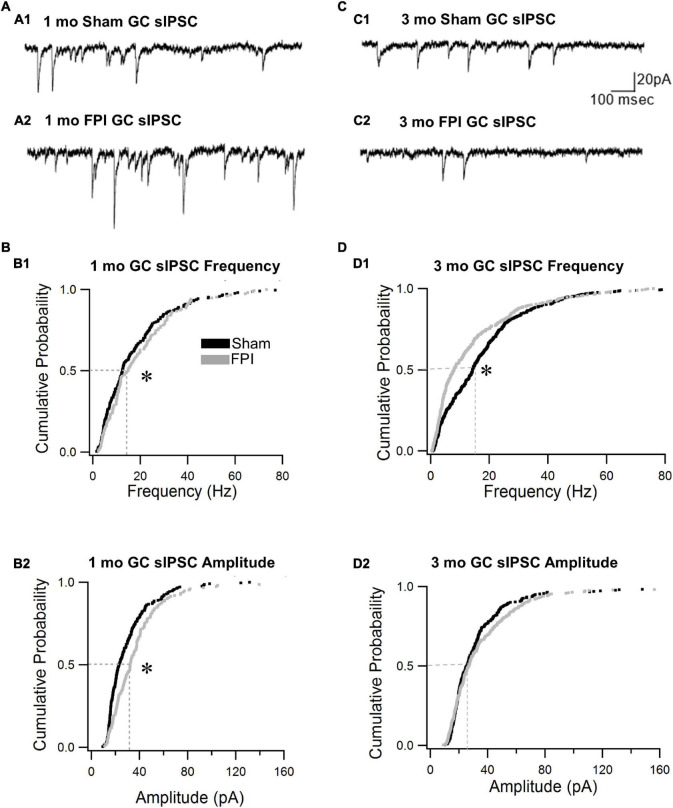
Granule cell spontaneous inhibitory postsynaptic current (sIPSC) frequency declines over time after brain injury. **(A)** Representative current traces illustrate sIPSCs in GCs from rats 1 month after sham **(A1)** and FPI **(A2)**. **(B)** Cumulative probability plots of sIPSC frequency **(B1)** and amplitude **(B2)** in granule cells 1 month after FPI. Dotted line illustrates the median. **(C)** Representative current traces illustrate sIPSCs in GCs from rats 3 months after sham **(C1)** and FPI **(C2)**. **(D)** Cumulative probability plots of granule cell sIPSC frequency **(D1)** and amplitude **(D2)** in rats 3 months after FPI. Dotted line illustrates the median. *Indicates *p* < 0.05.

We previously reported that the frequency of spontaneous inhibitory postsynaptic currents (sIPSCs) was increased in GCs 1 week after FPI both in the presence and absence of glutamate receptor antagonists (Gupta et al., 2012). To isolate the activity of the inhibitory circuit, we examined the effect of trauma on sIPSCs in the presence of the glutamate receptor antagonist kynurenic acid (3 mM). We find that GC sIPSC frequency and amplitude remained elevated 1 month after FPI (Figures 3A,B, frequency in Hz, sham: 17.6 ± 1.1; FPI: 19.4 ± 1.2, *p* = 7e^–9^ by WRT, Cohen’s d = 0.4; amplitude in pA sham: 30.1 ± 1.4; FPI: 38.5 ± 2.4, *p* = 0.0003 by WRT, Cohen’s *d* = 0.4, *n* = 9/5 sham and 6/5 FPI). However, sIPSC 10–90% rise time and amplitude-weighted decay time constant (τ_*decay*_) were not different from age-matched controls (10–90% rise time in ms, sham: 0.2 ± 0.01, FPI: 0.2 ± 0.03, *p* > 0.05 by *t*-test; τ_*decay*_, sham: 3.4 ± 0.1, FPI: 3.0 ± 0.3, *p* > 0.05 by *t*-test in *n* = 9/5 sham and 6/5 FPI). In contrast, GC sIPSC frequency was reduced 3–5 months after FPI (frequency in Hz, sham: 20.1 ± 1.6, *n* = 14/5; FPI: 12.7 ± 1.8, *n* = 10/6, *p* = 3e^–6^ by WRT, Cohen’s d = 0.2), while sIPSC amplitude was not different between groups (Figures 3C,D, amplitude in pA sham: 34.5 ± 1.9, *n* = 14; FPI: 40.3 ± 3.6, *n* = 10, *p* = 0.33 by WRT). Both 10–90% rise time and τ_*decay*_ of GC sIPSCs were not different between groups 3 and 5 months after FPI (10–90% rise time in ms, sham: 0.2 ± 0.01, FPI: 0.2 ± 0.013, *p* > 0.05 by *t*-test; τ_*decay*_, sham: 2.9 ± 0.1, FPI: 3.2 ± 0.2, *p* > 0.05 by *t*-test in n = 14/5 sham and 10/6 FPI). There was significant effect of age on sIPSC frequency, with sham controls showing a developmental increase in frequency [*F*_(2,32)_ = 23.5, *p* < 0.001], while sIPSCs frequency after FPI increased 1 week to 1 month followed by a decline over 3 months [*F*_(2,20)_ = 8.49, *p* = 0.002]. These data indicate simultaneous developmental- and injury-induced plasticity and suggest a decline in basal interneuronal activity 3–5 months after FPI.

### Long-Term Apparent Recovery of Semilunar Granule Cell Inhibition After Brain Injury

**FIGURE 4:**
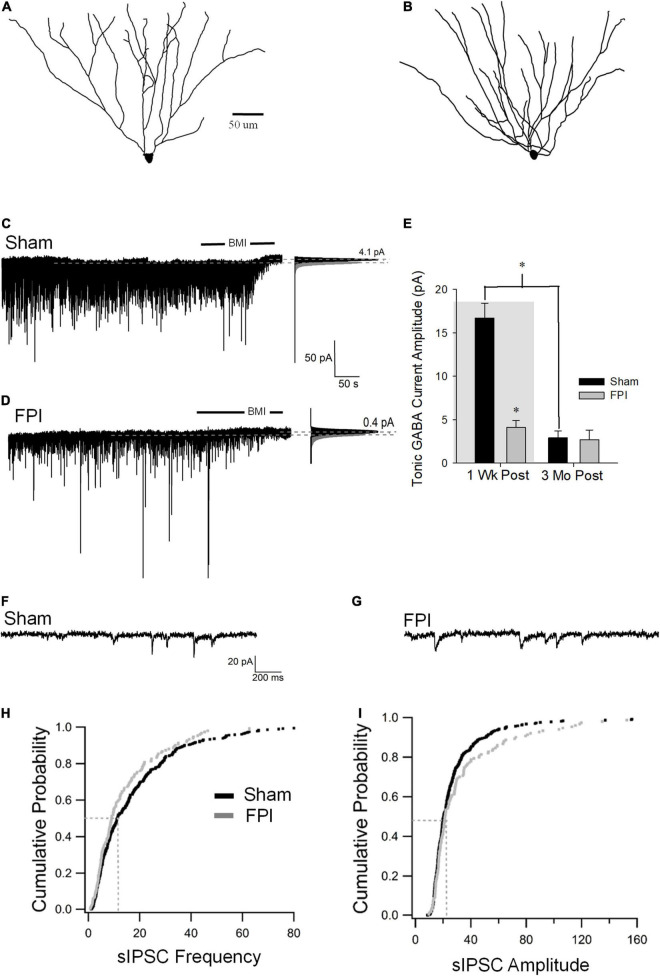
Long-term injury-induced changes in GABA currents in semilunar granule cells. **(A,B)** Representative Neurolucida reconstructions of semilunar granule cells from rats 3 months after sham **(A)** and FPI **(B)**. **(C,D)** Representative traces from semilunar granule cell 3 months after sham **(C)** or FPI **(D)** illustrate tonic GABA current as the baseline current blocked by bicuculline. Panels to the right show Gaussian fits to the positive half of histograms derived from 30-s recording periods in control conditions and during the perfusion of BMI used to determine tonic current. The dashed lines indicate the Gaussian means, and the difference in currents are noted. **(E)** Summary plot of tonic GABA current amplitude (pA) in SGCs from rats 1 week (data from Gupta et al., 2012 shown in gray box) and 3 months after FPI. **(F,G)** Representative current traces illustrate sIPSCs in SGCs from rats 3 months after sham **(F)** and FPI **(G)**. **(H)** Cumulative probability plots of SGC sIPSC frequency **(H)** and amplitude **(I)** in rats 3 months after FPI. Dotted line illustrates the median values. *Indicates *p* < 0.05.

SGCs undergo a marked decrease in amplitude of tonic GABA currents 1 week after FPI (in pA, sham: 16.7 ± 1.7, *n* = 8; FPI 2.9 ± 0.8, *n* = 9 *p* < 0.05 by *t*-test, previously reported in Gupta et al., 2012), which contrasts with the increase observed in GCs. Since SGCs show developmental reduction in tonic GABA currents (Gupta et al., 2020), we examined whether injury-induced differences in SGC GABA currents persisted to 3 months. Based on criteria established in earlier studies, SGCs were identified from biocytin fills based on the presence of multiple primary dendrites, semilunar somata, and wide dendritic span and somata located in the molecular layer (Figures 1D, 4A,B). SGC tonic GABA currents were not different between sham and FPI at 3–5 months post injury (Figures 4C,D, in pA, sham: 2.9 ± 0.8, *n* = 9/7; FPI 2.7 ± 1.1, *n* = 6/4 *p* = 0.8 by *t*-test). Interestingly, while there was an effect of age in sham controls (*F*_1,15_ = 58.2, *p* < 0.001), which is consistent with the developmental decline of SGC tonic GABA currents into adulthood (Gupta et al., 2020), there was no age-related change in SGC tonic GABA currents after FPI [*F*_(1,13)_ = 0.97, *p* = 0.34] (Figure 4E).

Similar to tonic GABA currents, SGCs also show a reduction in sIPSC frequency 1 week after FPI (in pA, sham: 15.6 ± 0.6, *n* = 6; FPI 4.8 ± 0.36, *n* = 6 *p* < 0.05 by *t*-test, previously reported in Gupta et al., 2012), which contrasts with the increase in GCs. However, sIPSC frequency in SGCs from rats 3 months after FPI was not different from age-matched sham controls (Figures 4F–H, in Hz, sham: 15.7 ± 1.2, *n* = 13/6; FPI 13.6 ± 0.8, *n* = 6/4 *p* = 0.08 by WRT). Additionally, sIPSC amplitude in SGCs was not different between FPI and age-matched sham controls 3 months after injury (Figure 4I, in pA, sham: 21.2 ± 2.0, *n* = 13/6; FPI 39.06 ± 9.4, *n* = 6/4 *p* = 0.08 by WRT). Although sIPSC frequency in SGCs from sham injured animals remained unchanged during this period [*F*_(1,17)_ = 0.003, *p* = 0.95], there was an apparent increase in sIPSC frequency in SGCs from 1 week to 3 months postinjury [*F*_(1,17)_ = 37.8, *p* < 0.001]. These data demonstrate differential temporal progression of changes in synaptic GABA currents in GCs and SGCs with a decline in inhibition overtime after injury in GCs and an apparent recovery of synaptic inhibition in SGCs.

## Discussion

TBI results in diverse cellular and network alterations (Morales et al., 2005; Pitkanen et al., 2009; Hunt et al., 2013; Neuberger et al., 2017a). Acute injury-induced death of GABAergic neurons in the dentate hilus initiates progressive changes in inhibition, which differ between injury models (Lowenstein et al., 1992; Toth et al., 1997; Hunt et al., 2011; Pavlov et al., 2011; Frankowski et al., 2019; Parga Becerra et al., 2021). Our data identify progressive changes in tonic and synaptic GABA currents, which differ between GCs and SGCs. Early post-FPI increase in tonic GABA currents in GC reported a 1 week (Gupta et al., 2012), persisted at 1 month, but was absent at 3 months. This decrease was due to a reduction in GC tonic GABA current after injury, while the amplitude in sham controls remained relatively stable over the same period. In contrast, the apparent recovery of early injury-induced reduction in SGC tonic GABA currents, reported previously (Gupta et al., 2012), by 3 months was largely due to a developmental decline in SGC tonic GABA current amplitude in sham rather than a recovery of the injury-induced decrease. Synaptic inhibitory events were examined in the presence of glutamate block to isolate the inhibitory circuit from glutamatergic plasticity (Santhakumar et al., 2001; Hunt et al., 2011; Folweiler et al., 2020; Korgaonkar et al., 2020b). We find that the early posttraumatic increase in sIPSC frequency in GCs, reported previously (Gupta et al., 2012), gives way to a significant decrease compared with age-matched sham controls by 3 months. This resulted from a delayed decline in sIPSC frequency 3 months after FPI. In SGCs, sIPSC frequency was not different from age-matched controls 3 months after injury largely due to a developmental decrease in SGC sIPSC frequency. Together, these data demonstrate complex, cell type-specific inhibitory plasticity after concussive brain injury in adolescent rats, which evolve alongside developmental plasticity.

Tonic and synaptic GABA currents are known to be altered after brain injury with outcomes varying between experimental models. GC tonic GABA current amplitude was reported to be similar to controls 2–20 weeks after murine CCI or severe FPI in adult rats (Pavlov et al., 2011; Boychuk et al., 2016; Parga Becerra et al., 2021), which contrasts with the increase we had reported 1 week after moderate FPI in adolescent rats (Gupta et al., 2012). Our current demonstration that GC tonic GABA current amplitude remains elevated 1 month after injury differs from that of Pavlov et al. (2011) and parallels our observation at 1 week (Gupta et al., 2012), while the recovery to control levels by 3 months is consistent with that of Pavlov et al. (2011). Since we adopted FPI in adolescent rats, when tonic GABA current amplitude shows a developmental peak during adolescence before declining to adult levels (Gupta et al., 2020), age at injury likely contributed to differences in the effect of injury on tonic GABA current amplitude observed in the two studies. Differences in injury severity and anesthesia could also contribute to the divergent effects. Consistent with prior work (Pavlov et al., 2011), the percentage increase in tonic currents induced by THIP was stable across all time points (not shown). It is possible that species or model-specific effects underlie the reduction in THIP modulation of GC tonic GABA currents after murine CCI (Boychuk et al., 2016; Parga Becerra et al., 2021). In keeping with interaction between developmental and injury-induced changes, the decrease in SGC tonic GABA currents observed 1 week post-FPI (Gupta et al., 2012) is eliminated by 3 months as a consequence of age-related decline in SGC tonic GABA currents in sham controls rather than recovery of postinjury changes. Indeed, it is possible that brain injury in adulthood, when the SGC tonic GABA currents are considerably lower than during adolescence, would not result in a discernable reduction in SGC tonic GABA currents. These results suggest that age of injury is a key factor in inhibitory changes in the dentate after moderate concussive injury. Specifically, concussive brain injury during adolescence may selectively reduce SGC tonic inhibition and alter feedback inhibition of GCs. Given the differences in posttraumatic pathology between the immature and adult brain (Saletti et al., 2019) and the high incidence of moderate concussions in adolescence, the selective perturbation of tonic GABA currents following concussion during adolescence could underlie heightened cognitive deficits in this population (Babikian et al., 2015).

Studies following both murine CCI and severe FPI in rats have reported reduced sIPSC frequency (Pavlov et al., 2011; Boychuk et al., 2016; Parga Becerra et al., 2021), which contrasts with the increase identified in our studies 1 week after moderate FPI (Santhakumar et al., 2001; Gupta et al., 2012). Since excitatory drive to interneurons can increase after injury (Santhakumar et al., 2001; Hunt et al., 2011; Folweiler et al., 2020), we examined the inhibitory circuit isolated in the presence of glutamate blockers. Interestingly, the postinjury increase in GC sIPSC frequency observed at 1 week (Gupta et al., 2012) was no longer evident at 1 month and reduced further, becoming significantly lower than age-matched controls by 3 months. In contrast, the decrease in SGC sIPSC frequency observed 1 week after injury was not present at 3 months, although the effect reflected developmental decrease in SGC sIPSC frequency in controls (Gupta et al., 2020) rather than recovery of sIPSCs after FPI. Since SGCs are proposed to drive polysynaptic lateral inhibition of GCs (Larimer and Strowbridge, 2010), it is possible that early reduction in SGC inhibition after brain injury may lead to increase in GC inhibition, impair memory processing, and delay epileptogenesis (Gupta et al., 2012; Folweiler et al., 2020; Korgaonkar et al., 2020a,b). It is possible that low SGC inhibition, together with progressive decline in GC inhibition after brain injury, contributes to the enhanced incidence of posttraumatic epilepsy following moderate concussive TBI in adolescent rats (Neuberger et al., 2017b; Korgaonkar et al., 2020b).

Tonic and synaptic inhibition are interrelated, with sIPSCs driving GABA spillover, which augments tonic GABA currents (Glykys and Mody, 2007). Consistently, we see that both synaptic and tonic GABA currents in GCs decline over time. However, the decrease in THIP-mediated currents suggests that with time after injury, an early increase followed by progressive reduction in expression of extrasynaptic GABA_*A*_R, including those containing δ subunits, contributes to the progressive reduction in tonic GABA currents after FPI. Moreover, SGC tonic GABA currents remained low at later time points, even when sIPSC frequency and amplitude after FPI were similar to controls, indicating that reduced extrasynaptic GABA_*A*_R expression likely drives changes in tonic GABA currents. The opposite direction of changes in sIPSC frequency in GC and SGCs is intriguing as it suggests that different populations of interneurons may innervate the cell types. While hilar somatostatin neurons undergo extensive cell loss, parvalbumin basket cells and molecular layer interneurons appear to survive (Toth et al., 1997; Santhakumar et al., 2000; Hunt et al., 2011; Frankowski et al., 2019; Folweiler et al., 2020) and may have enhanced contribution to GC sIPSCs after brain injury. Alternatively, the inhibitory network reorganization after brain injury may lead to cell-specific innervation, possibly as an attempt at restoring homeostasis at the level of cellular excitability. Additionally, since parvalbumin basket cells and molecular layer interneurons express GABA_*A*_R δ subunits (Glykys et al., 2007; Yu et al., 2013), it is possible that changes in interneuronal tonic GABA currents could influence their excitability and alter GC sIPSC frequency as observed in epilepsy (Yu et al., 2013). Indeed, recent data indicate that inhibition of dentate parvalbumin neurons may be increased after FPI (Folweiler et al., 2020).

In summary, we find progressive decrease in GC inhibition after concussive brain injury in adolescence, while developmental change in SGC inhibition drives the apparent normalization of SGC inhibition. Reduced SGC inhibition could enhance SGC recruitment during afferent inputs impairing its ability to shape input-specific lateral inhibition of GCs degrading memory processing following concussive brain injury. Progressive decline of the early increases in tonic and synaptic inhibition and eventual depression of sIPSC in GCs 3 months after brain injury could render the adolescent brain particularly vulnerable to impaired cognitive function and epileptogenesis observed over time.

## Data Availability Statement

The raw data supporting the conclusions of this article will be made available by the authors upon reasonable request, without undue reservation.

## Ethics Statement

The animal study was reviewed and approved by IACUC of Rutgers-NJMS and IACUC of University of California at Riverside.

## Author Contributions

AG performed the experiments. AG, LD, AP, and FE analyzed the data. AG and VS conceptualized, designed the research, and interpreted the results of the experiments. LD and AG prepared the figures. AG, LD, and VS drafted the manuscript. AG, LD, AP, FE, and VS finalized and approved the manuscript. All authors contributed to the article and approved the submitted version.

## Conflict of Interest

The authors declare that the research was conducted in the absence of any commercial or financial relationships that could be construed as a potential conflict of interest.

## Publisher’s Note

All claims expressed in this article are solely those of the authors and do not necessarily represent those of their affiliated organizations, or those of the publisher, the editors and the reviewers. Any product that may be evaluated in this article, or claim that may be made by its manufacturer, is not guaranteed or endorsed by the publisher.
